# Ictal and Peri-Ictal Psychopathology

**DOI:** 10.3233/BEN-2011-0314

**Published:** 2011-03-29

**Authors:** Marco Mula, Francesco Monaco

**Affiliations:** Department of Clinical and Experimental MedicineAmedeo Avogadro UniversityDivision of NeurologyUniversity Hospital Maggiore della CaritàNovaraItaly

**Keywords:** Epilepsy, post-ictal psychosis, post-ictal depression, per-ictal dysphoria

## Abstract

Patients with epilepsy may experience psychiatric symptoms preceding the seizure (pre-ictal), following the seizure (post-ictal), independently of seizure occurrence (interictal), or as an expression of the seizure (ictal). Compared to interictal, peri-ictal psychiatric symptoms are less investigated and recognized. However, they contribute substantially to disability and distress among people with epilepsy.

The relationship between interictal and periictal symptoms is still largely unknown but it seems that they are intimately related in epilepsy. Greater appreciation and understanding of the periictal period is clinically important, providing a model for understanding basic mechanisms underlying mood and thought disorders and the substrates of cognition, volition, emotion, and consciousness.

The present paper is aimed at reviewing major psychiatric symptoms that may occur around the ictus with special attention to clinical descriptions and relationships with interictal psychopathology.

## 1. Introduction

Patients with epilepsy may experience a number of psychiatric and cognitive symptoms or behavioral manifestations during the period around the ictus. They are obviously unique to patients with epilepsy and are referred to as peri-ictal symptoms [11,24]. These include symptoms preceding (pre-ictal) or following (post-ictal) the seizure or occurring as an expression of the seizure activity (ictal).

The practicality of classifying behavioral symptoms according to their temporal relation to seizure occurrence has been recognized for a long time (Fig. 1). These phenomena have been well described by Gowers [15] and Jackson [17] but also Kraepelin [26] and Bleuler [5] recognized their occurrence in epilepsy. However, among all behavioral manifestations, peri-ictal symptoms still remain the least recognized by clinicians and the least investigated in systematic research studies. Such paucity of data probably reflects the attention of clinicians to manifestations strictly connected to cortical EEG epileptic abnormalities, considering all manifestations occurring during an apparently "normal" or aspecific EEG as meaningless. In fact, ictal psychopathology has been better defined and described than peri-ictal phenomena. Nonetheless, peri-ictal psychiatric symptoms contribute substantially to disability and distress among people with epilepsy [6].

This paper is aimed at reviewing and discussing current knowledge about peri-ictal psychiatric symptoms in patients with epilepsy, with special attention to clin-

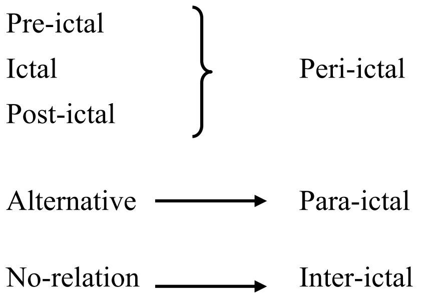

Fig. 1. Classification of psychiatric symptoms according to their temporal relation with seizures.

ISSN 0953-4180/11/$27.50 © 2011 – IOS Press and the authors. All rights reserved ical features and differential diagnosis with interictal psychopathology.

## 2. Pre-ictal symptoms

Pre-ictal psychiatric symptoms usually consist of cluster of symptoms preceding seizures of variable duration, ranging from a few minutes up to three days. Such symptoms, although not characterized by any detectable surface EEG change, probably represent the expression of underlying epileptic activity.

Around one-third of patients with partial seizures report premonitory symptoms, usually before secondary generalized tonic clonic seizures [13]. Prodromal moods of depression or irritability may occur hours to days before a seizure and are often relieved by the convulsion. Blanchet and Frommer [4] investigated the presence of pre-ictal psychiatric symptoms in 27 consecutive patients who were asked to rate their mood on a daily basis for a period of 1 month. Rating scales identified the presence of dysphoric symptoms, consisting of irritability and mood changes, approximately 3 days before the seizures. These symptoms worsened in severity closer to the time of the seizure and remitted approximately 1 day after the seizure, although in some cases symptoms persisted for up to 3 days after the seizure. A recently published study pointed out that behavioral changes are the most frequently reported pre-ictal symptoms, being characterized by irritability or decreased tolerance and lasting several hours [35].

Recently, a prevalence of pre-ictal dysphoric symptoms in patients with epilepsy has been reported. Around 13% of patients experienced irritability, dysphoria or depressed mood preceding seizures [29]. Such feelings are almost indistinguishable from interictal ones, apart from duration and close relation with seizure occurrence. It seems, therefore, important for clinicians to enquire about these phenomena, because they cannot be detected by rating scales or questionnaires [30].

## 3. Ictal symptoms

In the majority of cases, ictal psychiatric symptoms are very brief (they last less than 30 seconds), stereotyped, occur out of the context and are associated with other seizure-related phenomena such as subtle or overt automatism or post-ictal confusion of variable duration. These symptoms represent simple partial seizures which may be followed by alteration of consciousness as the ictus evolves to a complex partial seizure.

Ictal fear or ictal panic is the most frequently reported ictal psychiatric symptom. It can be a common manifestation of partial seizures originating in right mesial temporal lobe structures [16], representing a simple partial seizure or an aura followed by a complex partial seizure or a secondarily generalized tonic-clonic seizure. Fear is reported as an aura by 10%–15% patients with partial seizures [13]. It seems more common in women than men [7,38] and seems to have a poor prognostic value for surgery [12].

Ictal depression is the second most frequently reported ictal psychiatric symptom. Such mood changes include anhedonia, feeling of guilt and intense suicidal ideation. It occurs as part of an aura in approximately 1% of patients and more commonly in temporal lobe epilepsy [13].

Ictal psychoses may represent the clinical manifestation of an absence status (generalized non-convulsive status) or mostly a psychomotor status (complex partial status epilepticus) [31]. Patients who experience ictal psychoses may present with either a prolonged twilight or confusional state (continuous form), or a series of recurring seizures with partial responsiveness between seizures (cyclic form) [39]. The duration of symptoms varies from hours to days and EEG shows focal epileptic abnormalities. Still, it is recognized that ictal psychoses of temporal lobe origin are characterized by a more profound impairment of consciousness as compared to extra-temporal ones where speech disturbances and confusion are fluctuating [39].

Ictal hallucinations are usually visual (less frequently auditory). Compared to psychotic hallucinations patients are aware that such symptoms reflect unreal phenomena. It has been stated that ictal hallucinations rising from the insular cortex are associated with a strong autonomic component. Devinsky et al. reported a peculiar type of ictal hallucinations, namely autoscopic or out of the body experiences [8]. The authors pointed out that in the majority of cases they have a localizing value (86% from the temporal lobe) but with no relevance for lateralization.

## 4. Post-ictal symptoms

Post-ictal symptoms may begin either immediately after the seizure (immediate post-ictal period) or more characteristically from 12 to 120 hours after a seizure or a cluster of seizures (also known as lucid interval).

Post-ictal psychoses are the best characterized phenomena among all peri-ictal manifestations. Historically, it was accepted that patients could develop psychoses in the context of the post-ictal state, the features of which were often manic or hypomanic, although more generally the presentation was one of a mixed mood episode with psychotic features. Original descriptions date back to Esquirol, Jackson and Gowers, however, post-ictal psychoses did not become the focus of attention until much later [28,34]. They represent the most frequent psychiatric manifestation in clinical practice accounting for as much as 25% of cases of psychosis in one early epilepsy series [9]. Variables linked to post-ictal psychoses in different case series can be summarized as: delay between the onset of psychiatric symptoms and the time of last seizure; relatively short duration of symptoms; strong affective component in the phenomenology; secondary generalized tonic-clonic seizures preceding the onset of post-ictal psychosis; long duration of the epilepsy; a prompt response to very low dose of antipsychotic drugs and benzodiazepines [10,21].

Oshima et al. [33] pointed out that post-ictal psychoses should be subdivided into two different subtypes: the nuclear type, with an established clinical picture and the presence of the lucid interval, and the atypical peri-ictal type, usually without the lucid interval and with quite a polymorphic clinical presentation. The former probably represents an indirect aftereffect of seizure activity whereas the latter is a direct manifestation of limbic discharge [33]. Kanemoto et al. [18] stressed the typical clinical phenomenology of post-ictal psychosis which seems to be characterized by grandiose delusions as well as religious delusions in the setting of a markedly elevated mood and the feeling of mystic fusion of the body with the universe. In addition, post-ictal psychoses seem to exhibit few schizophreniform psychotic traits such as perceptual delusions or voices commenting, and well-directed violent and self-destructive behavior seem a specific hallmark [20]. Post-ictal psychosis appeared to be a predictor of bilateral ictal foci [22] and consequently a poor surgery outcome [19].

Post-ictal mood changes are less recognized in psychiatric practice as compared to post-ictal psychoses. However, they can be frequently reported by patients and relatives. A case series in a monitoring unit reported 18% of patients had at least five symptoms of depression lasting more than 24 hours but less than 2 weeks (required by DSM-IV for a major depressive episode) [25]. A comparison of seizure-related variables between these subjects and patients without any post-ictal psychiatric symptom failed to reveal any differences, although patients with post-ictal depression seem to be more likely to have a previous history of psychiatric disorders [24].

Manic/hypomanic symptoms are reported in 22% of patients, often with associated psychotic phenomenology [25]. It seems that post-ictal mania has a distinct position among mental disorders observed in the post-ictal period. Such manic episodes last for a longer period and have a higher frequency of recurrence than post-ictal psychoses, being associated with an older age at onset, EEG frontal discharges and dominant hemisphere involvement [32].

Post-ictal anxiety is reported by 45% patients [25]. The median duration of symptoms ranges from 6 to 24 hours. In one third of cases, post-ictal anxiety may last 24 hours or longer. In about 33% of cases post-ictal anxiety is reported by patients with a previous history of an anxiety disorder.

## 5. Para-ictal symptoms

Among all psychiatric manifestations that may occur around the ictus those associated with seizure control most need to be taken into account. Landolt reported EEG investigations of patients with epilepsy and paroxysmal psychiatric disorders, describing a group of patients who had productive psychotic episodes with "*forced normalization*" of the EEG. In other words, abnormal EEGs of these patients improved or normalized during the time that they were psychotic [27]. Subsequently, Tellenbach [37] introduced the term "*alternative psychosis*" for the clinical phenomenon of the reciprocal relationship between abnormal mental states and seizures, which did not, as Landolt's term did, rely on EEG findings. In any case, since the early observations of Landolt, a number of patients with alternating psychosis have been documented to put their existence beyond doubt and an association with the prescription of antiepileptic drugs was noted [40]. In fact, in many of the series described, the precipitation of the abnormal behavioral state or the psychosis has been linked with the prescription of anticonvulsants but it is important to note that this phenomenon should not be restricted to drug-induced seizure control. In fact, it is likely that in patients who develop de novo psychosis following epilepsy surgery, forced normalization may play such a role, and a case of an alternative psychosis secondary to vagus nerve stimulation has been documented [14], suggesting that the mechanisms underlying the control of seizures are strictly interlinked with those of psychosis.

As Landolt himself pointed out, there is no specific clinical presentation of this phenomenon [27]. Of 44 clinical episodes of forced normalization described by Wolf [36], the commonest syndromes were psychosis but in nine cases pre-psychotic dysphoria was described, characterized by insomnia, restlessness, anxiety and social withdrawal. In another two cases there were dysphoric episodes and true manic episodes was also described in two other patients. Wolf made the point that the symptomatology was often determined by personality structure, psychiatric history or familial predisposition.

## 6. Relationship between peri-ictal and interictal symptoms

Relationships between interictal psychopathology and peri-ictal behavioral manifestations are poorly specified. However, emerging evidence clearly suggests that these variables are intimately related, with the majority of supportive data coming from the literature about psychoses in epilepsy.

On one hand, interictal symptoms represent a risk factor for the development of peri-ictal psychiatric symptoms. In fact, Alper et al. [3] showed that a previous history and a family history of any psychiatric disorder may be associated with the development of post-ictal psychotic episodes. On the other hand, post-ictal psychopathology seems to be connected with the development of a chronic psychiatric disorder. It has been estimated that around 13.9% of patients with a history of post-ictal psychosis may subsequently develop an interictal psychosis [2,23]. Adachi et al. demonstrated that, in the majority of cases, post-ictal psychotic episodes last less than 1 month but they can be prolonged when individuals have an underlying vulnerability to psychosis [1].

Another interesting issue is that of peri-ictal exacerbation of interictal symptoms. A U.S. study reported exacerbation in the post-ictal period in 94% of patients and de-novo post-ictal symptoms with different psychopathological features in 83%. Interestingly, the authors pointed out that antidepressant drug treatment did not prevent the development of post-ictal mood symptoms but was shown to be effective for interictal symptoms [11]. Interestingly, it has been suggested that antiepileptic drugs with negative psychotropic properties (e.g. barbiturates and benzodiazepines) may yield a trend toward a greater likelihood of developing post-ictal exacerbation of interictal symptoms [11].

## 7. Conclusions

The presented data illustrate the relatively high prevalence of peri-ictal psychiatric symptoms and their very close relationship with interictal ones. Without doubt, the recognition of these symptoms represents the essence of a thorough appreciation of psychiatric phenomena in epilepsy, including (as it pertains to) the interpretation of its response to treatment. In fact, the failure in identifying such conditions has several practical implications in terms of prognosis and therapeutic strategies.
